# Fertility Preservation in Female Cancer Patients

**DOI:** 10.1155/2012/695041

**Published:** 2012-10-20

**Authors:** I. Demeestere, O. Basso, F. Moffa, F. Peccatori, C. Poirot, E. Shalom-Paz

**Affiliations:** ^1^Research Laboratory on Human Reproduction and Fertility Clinic, Department of Obstetrics and Gynecology, Universite Libre de Bruxelles (ULB), Hôpital Erasme, 1070 Bruxelles, Belgium; ^2^Department of Epidemiology, Biostatistics, and Occupational Medicine, McGill University Health Centre, Royal Victoria Hospital, Montreal, QC, Canada H3A 1A1; ^3^Assisted Reproduction, Gynecology and Obstetrics Clinic, Instituto Marques, 08034 Barcelona, Spain; ^4^Fertility & Procreation in Oncology Unit, Department of Medicine, European Institute of Oncology, 20141 Milano, Italy; ^5^UF de Biologie de la Reproduction, Groupe Hospitalier Pitié-Salpêtrière, 75013 Paris, France; ^6^Department of Obstetrics and Gynecology, McGill University Health Centre, Royal Victoria Hospital, Montreal, QC, Canada H3A 1A1

Thanks to the progress in oncology research, the cure rate of patients with cancer has greatly improved, reaching 70% or higher in children and adolescents. Consequently, the long-term side effects of the treatment, including chemotherapy-induced premature ovarian failure, have received broader attention. The prospect of definite infertility can induce major psychological distress in cancer patients, as the inability to reproduce can represent a profound loss for women, affecting their self-esteem as well as their relationships. During the last decade, information and management of fertility issue before oncological treatment have become part of the guidelines that should be considered by all oncological units. Fertility preservation is, however, an area that needs specific attention and knowledge by dedicated physicians working in this field. Therefore, collaboration between oncological units and fertility clinics is necessary to adequately counsel each patient with a short delay. 

The papers in this special issue have examined the impact of chemotherapy and radiotherapy on ovarian function and reviewed the different fertility preservation strategies in light of the current clinical and experimental advances. For the occasion, 8 papers were accepted for publication, dealing with the different approaches to preserve fertility, with a special attention on children and adolescents. 

The first step of fertility preservation management is the evaluation of the individual risk of premature ovarian failure according to the patient's age and type of treatment. Many factors affect the risk of injury to the ovaries, including the specific chemotherapeutic agents, the cumulative dose, and whether or not radiotherapy is administered. These issues were discussed by different authors, and specifically addressed for children by S. Gnaneswaran et al. According to the potential level of gonadal damage, different options to preserve fertility can be proposed. A review article by some of the editors of this special issue discusses the different approaches to preserve fertility, focusing on their advantages and limitations. Vitrification of embryos is a well-established procedure and is currently performed in many IVF laboratories with a high success rate for adults. However, not all cancer patients can benefit from stimulation protocols for embryo and, more recently, for oocyte freezing. The effectiveness of slow freezing versus vitrification techniques for oocytes storage is well reviewed by A. Revelli et al. Cryopreservation of ovarian tissue offers an attractive alternative for many patients. The experience in pediatrics patients of the Orsola-Malpighi Hospital in Bologna was reported by R. Fabbri et al. J. Michaeli et al. report new revised guidelines for providing ovarian cryopreservation to premenarcheal girls with cancer and address the medical and ethical aspects of the procedure.

The safety of autotransplantation of cryopreserved ovarian cortex remains, however, a major concern for both adults and children undergoing the procedure. The paper of L. Bockstaele et al. discusses this important issue, focusing on the main tools for detecting disseminated cancer cells currently available, their limitations, and clinical relevance. 

Future options related to the possibility of postnatal neogenesis in the ovary are subjected to many debates in the literature and discussed in the paper by D. Bhartiya et al.

Finally, the risks associated with conceiving after cancer were addressed by M. L. Matthews et al. in a review discussing also the different aspects of fertility preservation management.

Through these 8 papers, the issue aims to provide an overview of the different available approaches to preserve fertility before oncological treatment in female adults and children.

